# Cognitive gene enhancements and the capitalist meritocracy

**DOI:** 10.1186/s12910-026-01377-8

**Published:** 2026-01-09

**Authors:** Sinead Prince

**Affiliations:** Centre for Biomedical Science, Yong Loo Lin School of Medicine, National University of Singapore, Singapore, Singapore

**Keywords:** Enhancements, Ethics, Cognition, Gene editing, Autonomy, Oppression

## Abstract

The relationship between cognitive enhancements (CE) and human autonomy or authenticity is generally positioned as *how* CE impact human autonomy or authenticity. But rarely, if ever, do we consider whether the value and pursuit of CE is an authentic one. In this paper, I will argue that the moral permissibility of cognitive *gene* enhancements is undone by the legitimate concern that the near universal value for such modifications is likely driven by oppressive norms for superintelligence and productivity. I argue that these norms derive from the capitalist meritocracy: an economic system that structures inclusion and success based on patriarchal and racist norms of intelligence and productivity. The claim that the use of such enhancements fits within the autonomous scope of individual power is thus far weaker than it claims to be, particularly within the context of genetic modification.

## Introduction

There is a long-standing debate in enhancement ethics as to the conceptual and normative nature of the relationship between cognitive enhancements (CE) and human agency, autonomy, and authenticity. Some argue that CE do not *negatively* affect autonomy. For example, DeGrazia [1] has argued that “it is far from self-evident that changing “core” traits is morally problematic if a person autonomously consents to their alteration.”^1^ Some go further to argue that CE can help us be *more* authentic or autonomous. For example, Bublitz and Merkel [5] have argued that insofar as authenticity is *existentially* conceived – the view that we create ourselves according to our values, rather than discover some “true” self – cognitive enhancements can help us live up to our authentic selves. Rueda et al. [6] have argued that genetic enhancements could enhance a person’s capability for procedural autonomy, namely, self-reflection and rationality. Similarly, Juth [7] argues that, “[i]n general, plans require capacities in order for them to be put in effect and enhancement technologies can increase our capacities to do the things we need to do in order to effectuate our plans”. Schaefer et al. [8] argue that genetic enhancements improve a person’s capability for reasoning, and thus contribute to human well-being by improving a person’s capacity for self-determination and choosing a life plan.

Conversely, some have raised concerns that CE undermine human authenticity. For example, Bandeira and Lenine [9] have argued that adopting a relational approach to autonomy, “cognitive enhancement is socially influenced by how individuals perceive themselves being affected by the decision to use enhancers.” For example, because there may be unequal access to genetic enhancements [10], the desirability for enhancement may not be because the person wishes to live in accordance with their ideals, but simply to *not fall behind in an unequal socioeconomic environment.* Elliot [11] also argues that the desirability for enhancements is to remedy the kind of shame that comes from not belonging to specific and damaging norms. Lewis [12] argues that enhancing cognitive capacities does not necessarily improve one’s capacity for autonomy, and that external influences, such as oppression or socialization, can undermine a person’s authenticity when considering the value of enhancements.

This debate, given how old its origins and conceptually diverse its contents, at times seems to simultaneously diverge and conflate concepts. I believe we ought to separate the question of whether enhancing oneself undermines or validates their authentic self, from the question as to whether the *value* or *desire* for enhancements is an authentic one [122]. Answering the latter is a precondition to the first. Particularly because in cognitive-genetic enhancements (‘CGE’), we are enhancing future persons who do not experience a *pre-* and *post*-*enhancement self.*

Why then, do people want to CE? We might respond, ‘well, wouldn’t being smarter be better?’ But we ought to ask whether this categorical presumption is the because intelligence is an objective good or something more pernicious. I argue the latter; much of the value vested in cognitive gene enhancements is rooted in the belief that *superior* intelligence is valuable or necessary for human well-being. I argue that this belief predominantly arises from oppressive norms of value within capitalist meritocracies (although my argument applies more broadly to any society that uses intelligence as a positional good by which people become deserving of resources). I first briefly define several modalities of CE. Second, I define relational autonomy, as well as the relationship between oppressive systems and inauthenticity. I then argue that several key features of the capitalist meritocracy, namely, the hierarchy of power based on racist and patriarchal norms of knowledge, credentialism, and capitalistic goals of productivity, together form an oppressive nexus in which we are manipulated into valuing a very specific concept of cognitive enhancement.^2^ As such, I conclude that there is good reason to believe that under these conditions, society does not pursue certain cognitive gene enhancements *authentically*. I then make final remarks about what this might mean for future discussions on the moral permissibility of cognitive gene enhancements.

The scope of this paper is necessarily limited. I aim only to draw conclusions about the impact of our current socioeconomic structures on our autonomy by analysing our preferences for cognitive gene enhancements, particularly those that we already use for ourselves. As such, I will first analyse the use and preferences for CE and apply this to genetic enhancements. I am also aware that cognitive *gene* enhancements (CGE) will most likely be sought out by parents for their children, and within this context, a whole range of additional issues arises, such as the rights and duties of parents to enhance their children. However, this is a separate debate, and I cannot do this debate justice here, but insofar as parents have preferences or values for particular cognitive gene enhancements, my claims about authenticity and autonomy will also apply to such contexts.

## Cognitive enhancements

CE are the modality of technologies that aim to improve capacities such as memory, attention, and executive functions, in individuals considered healthy [13]. One modality is pharmaceutical agents and includes prescription stimulants like methylphenidate (Ritalin) and modafinil that are used off-label by healthy persons aiming to improve focus, alertness, planning abilities, decision-making, and learning efficiency [14]. The prevalence of the use of CE by healthy people ranges widely from 2% to 45.6% of international samples [15–18].

Another modality is mechanical devices. These include neurointerfaces, implantable brain chips, AI-driven devices, and other brain-computer interfaces (BCIs). These aim to establish direct communication pathways between the brain and external device to enhance memory capacity, processing speed, or even facilitating novel forms of sensory experience.^3^ Some of these goods are available direct to consumers and are predicted to form a highly profitable market [20].

Genetic modifications represent a more prospective, and indeed more ethically contentious, avenue for CE, centred on the alteration of brain growth factors or other genetic determinants of cognitive function. These include the modification of genes critical to neurodevelopment, synaptic plasticity, or the synthesis of neurotrophic factors vital for neuronal proliferation, survival, and differentiation [21]. For example, the manipulation of brain-derived neurotrophic factor (BDNF) pathways, known for their crucial involvement in learning and memory processes, might theoretically enhance these cognitive domains [22]. Currently, no empirical evidence suggests this is possible with humans, particularly because cognition is polygenic, and most experimental evidence of successful outcomes coming from research on animal models [23–26]. Nevertheless, our understanding of the genetics of cognition are ever improving, as are our means of genetic modification improving with versatility and precision, such as recent advances in prime editing [27–29].

Many of the existing arguments in this debate and consequently paper pertain to those making decisions to use CE on themselves, such as adults consenting to the use of DBS or pharmaceutical agents. However, the purpose of this paper is understanding whether the pursuit of cognitive *gene* enhancements is an authentic one, and this is necessarily constrained to genetic modification of embryos at the request of the intending parents. Genetic modification of embryos comes with a whole host of other ethical issues, such as equality,^4^ safety, risk, and the moral limits of parental control over their child.^5^ I will nevertheless limit the scope of this paper to the *social* pursuit of cognitive gene enhancement, that is through the research, development, and regulation of this technology by society because we *value* such enhancements. Although, questions of whether societal pursuit of cognitive gene enhancements are generally authentic will nevertheless apply to parental pursuit of embryonic enhancements.

## Autonomy

The principle of autonomy is the view that people ought to be self-determinate about decisions pertaining to them, and particularly, their bodies [37]. There is naturally debate about its precise conditions and its moral limits, but there is widespread agreement that, whatever it is, autonomy is intrinsically important for respecting a person’s dignity or capacity for rationality. Under liberal notions of autonomy, there are several conditions seen as essential for a person to have autonomy or make an autonomous decision: competency, sufficient information, a meaningful range of options, and freedom from undue influence or voluntariness [38]. The latter is of most relevance to discussions on CE.^6^

Voluntariness is rooted in the ideal of authenticity: it is about ensuring that a person’s decision is truly theirs and that they were not forced, manipulated, or unduly influenced into such a decision or choice [39].^7^ Relational theories of autonomy can help us distinguish legitimate from illegitimate influences.^8^ These theories argue that autonomy is a relational concept – upheld and undermined by the relationships who have power over us, such as our families, friends, and partners, and doctors, employers, and social systems at large [40–42]. These theories legitimise the dependence of humans on each other to make decisions and form our values, but also, to exist more generally [43]. Humans are relationally constituted, and our sense of self and how we gradually form our identities over time is dependent on the systems and structures in place as we develop our values. In order to autonomously make decisions, we need self-trust and self-respect, which are inherently dependent on our relationships and social structures [44]. For example, to know whether a woman is autonomously consenting to cosmetic surgery, we need to take into account her surrounding social structures and systems of value, such as the extent of patriarchal influences on her decision-making. Our beliefs are not formed in vacuums; what matters for voluntariness is therefore whether a person affirms the decision-making process in which a person formed their values, rather than ensuring the person was free from influences altogether [39].^9^

Distinguishing whether our societal norm for cognitive gene enhancements is a legitimate, and therefore autonomy respecting, influence therefore comes down to the presence of *manipulation* [5, 12]. Manipulation is the intentional alteration of facts, emotions, or circumstances so to control behaviour [53]. Manipulation can be environmental, in the way that our choices or options are systematically narrowed by others, such as through threats of violence, or through unjust consequences if we fail to conform [54]. Manipulation can also be psychological, insofar as it is an attempt to bypass human rationality to control behaviour [53, 55, 56]; a move seen as *blameworthy* because it exploits our emotional vulnerability (generally fear) and ignores our agency and capacity for rationality. This form of manipulation can be rapid, or a process brought about by socialisation that denies us an opportunity for critical reflection throughout the value formation process [39]. Cudd [57] defines this form of manipulation as when “one is oppressed through one’s mental states, emotionally or by manipulation of one’s belief states, so that one is psychologically stressed, reduced in one’s own self-image, or otherwise psychically harmed”. These cases are most identifiable by analysing the objective value of the decision or choice, namely, the person holds values for which they lack reason to value [58]. For example, when people in oppressive relationships internalise values, such as low self-worth due to their appearance, that are not in their own best interests.

Oppressive systems are widely accepted as a common and insidious form of manipulation [57, 59–61]. These are systems that use unjust discrimination, norms, and systems to control and maintain dominance over groups of individuals. This is why relational theories implore the importance of understanding existing social structures in evaluating autonomy in a decision-making process. Because sometimes it’s not always a particular individual who is manipulating someone into holding a particular value, but a societal system constructed so to undermine a person’s self-respect, self-worth, and self-trust and thus control their behaviours [44]. Lewis [12] argues that “internalized oppression and socialization into overly paternalistic, demeaning or unjust interpersonal and social practices can all compromise an agent’s power to exercise her autonomy”.

Oppression manifests in many forms. Young [62] explores several “faces of oppression”, of relevance here is marginalisation, powerlessness, and cultural imperialism. These lenses can be used to critically examine the ways in which people are oppressed into desiring certain goods, such as valuing certain norms or exerting particular behaviours. In this paper, I will examine the ways in which cognitive gene enhancements can be valued or pursued for inauthentic and therefore nonautonomous reasons, namely, because such values arise from oppressive norms that exist in our social structures i.e., in a capitalist meritocracy.^13^

## The capitalist meritocracy

A meritocracy is a system of valuation by which employment and economic success is ranked in a hierarchical system according to aptitude and effort for the purposes of rewarding the most qualified. Meritocracy originated from an overturning of aristocratic nepotism: we shouldn’t allocate jobs and rewards based on who our parents are or where we come from, but whether we are qualified for the job. A meritocracy, therefore, is meant to be just because it rewards merit rather than favouritism or luck - something we have control over rather something we do not [63]. Those who succeed are therefore justified in feeling confident in their success and owning their rewards because they *deserve* their success. Those who rank much lower consequently only have themselves to blame.

In this section I will first argue that we internalise norms of cognitive superiority as justification for our meritocratic rank. Second, that we subsequently value CE to help us achieve these norms. Third, that the meritocracy is *oppressive* and therefore is illegitimately influencing us. I will present three representative and common examples of meritocratic capitalist values that are relevant to CE to illustrate these arguments: standardisation tests in school, credentialism, and the capitalist division of labour according to capacity for productivity.

### Intelligence and cultural imperialism

Standardisation tests in schools and universities are intended to be objective assessments of the students’ cognitive capacities and problem-solving skills to provide entry to further education or entry-level jobs based purely on merit. However, as merit is a system of value, the definition of excellence or value is defined by the cultural norms that systematically underpin scholastic institutions. In many modern societies, these norms are both racist and patriarchal in origin and practice [62], resulting in the imperialization of cognitive excellence as abstract rationality.

The racist origins of standardisation or ‘IQ’ testing are relatively uncontroversial. These tests were developed by Alfred Binet and Henry H. Goddard during the 1900s eugenics movement to “objectively” validate the existing class hierarchies on the basis that persons of colour are less talented and biologically advanced than those of Nordic heritage, and thus deserving of segregation and domination [64–67]. Despite changes in the 1980s, researchers argue that this standardisation testing used in schools “served the interests of Whiteness and homogenization while actively working against the interests of diversity and multicultural education” [68, 69]. This is because standardisation tests measure the knowledge valued by white people; they exclude and reject the lived experience of people of colour, which *informs* their knowledge and understanding. For example, Australian First Nations’ children have rich cultural competence that is ignored by mainstream education and testing [70], and Aboriginal English is often perceived as a “bad” version of Standard Australian English [71]. Whilst schools cannot test all knowledge, they systemically only teach, test, and *rank* students according to racially biased knowledge and the skills preferred by colonial and capitalist traditions, such as mathematics, statistics, science, and literature [72].

Au [68] argues that the mandated and near universal use of tests to allegedly objectively rank students not only measures, but *reinforces*, wealth and racial inequalities. For example, children of colour generally experience significantly worse academic rankings than white children [70, 73]. Even within families with high socioeconomic status, in comparison to white students, Black students are more likely to attend schools with higher poverty rates, less experienced educators, and lower graduation and college enrolment rates [73]. Research suggests that race and socioeconomic status account for over 50% of variance in standardisation test scores in language and math [74]. States then use these poorer outcomes from racially diverse students to justify differentially investing in their education and opportunities: between 2001 and 2012, schools in Chicago with low testing scores were closed down, resulting in 85% of the students affected by closures being Black despite only 40% of the entire state student population being Black [66]. Standardisation testing does not measure cognitive intelligence, but race (amongst other factors including wealth [75]), even though such testing is then used to measure student eligibility for essential opportunities like tertiary education, jobs, and scholarships. This is not merit, but injustice. 

The kind of intelligence measured in standardisation testing is also largely patriarchal. Young (1990) argues that societal norms of intelligence are untouched of “material embodied elements”, kept pure from the feminine: relational, emotional, and social. Intelligence, as the default concept, is masculine: objective, abstract, rational, and we have professionalised and privileged professions that require this virtue.^10^ The default concept of intelligence is the masculine – other types of intelligence require the qualifying adjective e.g., emotional intelligence. Lindemann [76] argues that this is because Kantian moral philosophy bases respect for persons on the ground that all persons are rational, and this intellectual virtue is privileged because it is prized by the patriarchy. As such, Young [62] argues that being intelligent is deemed appropriate for white men, while knowledge and skills that centre on caring for bodies and emotions is stereotyped as feminine and people of colour. Jagger [77] describes this as “normative dualism”, in which work is hierarchical, distinguishing the value of intelligence as distinct from the body. Young questions this norm, asking whether doctors are really more “intelligent” than librarians and plumbers; whether disciplining, playing with, and teaching young children requires less intelligence, and therefore deserves less socioeconomic reward than the kind of intelligence manifested in stock brokers; and whether determining how to move bulky furniture in tiny apartment stairwells without damaging a product is less admirable than computer software programmers. The normative hierarchy of masculinity as *intelligent* and feminine as emotional divorces the mind from the body through unfair and unjust distribution of rewards: deeming those who perform well on masculine measures as objectively intelligent, and all Others as undeserving of equal rank and social status.

Although not inherently racist or patriarchal, in reality, standardisation testing and scholarly institutions have become the social structures that measure and systematise intelligence as white and masculine.^11^ Young [62] terms this cultural imperialism: the “universalisation of a dominant group’s experience and culture, and its establishment as the norm”. Young argues that the systemic racism and patriarchy in our cultural norms result in structural class differences, the injustice of which is how “[p]restige, which is entirely a cultural and symbolic construct, then permits differential material reward and privilege” to white masculine work. Katz [66] argues that “[t]he quest to pin down intelligence has always served imperial and capitalist institutions by producing such hierarchies of human worth.” Failing to succeed according to this type of intelligence can have significant economic and social consequences, which leads to the situation where we *must* conform or face significant consequences i.e., manipulation [46].

### Credentialism and powerlessness

Meritocracies confer credentials on those who deserve them and employers value credited employees because they have been independently verified as valuable. Credentialism is a system of meritocratic justification using qualifications. In our society, college and university degrees are the way such qualifications are quantified. This can be in our best interests; we need surgeons to be trained in how to perform surgery, engineers to know what they’re doing when building skyscrapers, and schoolteachers trained in child learning and development.

However, *in our society*, credentials are social constructs and those who lack such credentials are not only ranked as socially and economically inferior but are powerless to address their disadvantage. Our meritocratic society requires those with the most merit to lead and hold positions of power, and when merit is proven through qualification, society becomes oppressively technocratic. For example, in 1901, only 15% of MPs in Australian Parliament held post-secondary qualifications [78], whereas in 2021, 76% of Australians MPs had at least one undergraduate degree (which is more than double the rate among everyday Australians), 95% of US House of Representatives had a degree, and 85% of the UK House of Commons politicians had a degree [79]. One Forbes study found that 34.6% of top Fortune 100 CEOs have at least a master’s or a doctorate degree [80], and another study found that globally, 98% of CEOs have at least a Bachelor’s degree, with 64% holding a master’s degree [81, 82]. Most positions of power mandate through recruitment some form of degree: managers, doctors, engineers, teachers, nurses, paramedics, researchers, accountants, pilots, pharmacists, dentists, lawyers, therapists etc. Those that don’t *require* degrees nevertheless competitively *screen* for degrees resulting in the highest median weekly earnings being managers and professionals [83].

Even when credentials do not train a person for a specific role, our society perceives those with credentials as having intrinsic merit. We believe that “any degree is better than no degree” [84]. Sandel [63] argues that the cultural insistence that “social and political problems are best solved by highly educated, value neutral experts is a technocratic conceit that corrupts democracy and disempowers ordinary citizens” and that this “notion that ‘the best and the brightest’ are better at governing than their less credentialed fellow citizens is a myth born of meritocratic hubris.” Katz [66] argues that this “[m]eritocratic sorting is bound up with judgments, about earning and deserving. These are inescapably public judgments, about whose talents and achievements are worthy of honor and recognition.” In a meritocracy, we believe that credentials warrant power, and those lacking credentials are thus deserving of their powerlessness.

Credentialism has also created a monopoly over income security, economic freedom, and the pursuit of meaningful work [85]. Those with non-school qualifications generally have the lowest incomes (e.g., hospitality, the arts, and recreation services) and less access to employment or full-time employment [86]. In one 2020 study, 67% of job applications explicitly required some form of college degree [87]. Some predictions estimate that 90% of the jobs created over the next five years will require a post-secondary qualification and at least 50% will require a higher qualification [88]. We use credentials to justify the division of labour according to accreditation and those with qualifications and intelligence are rewarded with higher paying, secure, safer, and respectable jobs in society [62]. In many countries, such qualifications and credentials are not free. Furthermore, even when free or low-cost, achieving such credentials takes time - time away from earning income, leaving the pool of those feasibly positioned to undertake credentials as those already enjoying socioeconomic privilege. As such, those without credentials also lack autonomy in the day to day; they cannot progress without credentials and they cannot achieve credentials without privilege.

This systematic powerlessness is structural: capitalism works to confer the power, wealth, and status of the energies and labour of the workers onto their superiors, continuously and permanently, without reciprocation (Young 1990, 218). This is the reality for most people suffer; most people work for necessity rather than personal fulfillment [89, 90]. As such, Veltman [90] argues that in a capitalist market, which at its core is a competitive economy, “employment is a limited good”, and so people are *coerced* into, rather than autonomously pursuing occupations that offer only powerlessness. This problem is worsening. Olsen (1983) argues that as institutions monopolise services and jobs through credentialing tools, few people are let into competitive institutions to get credentials, and fewer graduate with employment opportunities. People without credentials cannot exercise their knowledge and capacities due to government restrictions on credentialed services. However, the meritocracy creates the false belief that this inequality is *just* because it is impartial and based on objective merit – not everyone can have every job. But allocating jobs according to merit does not reduce inequality. Instead, we use meritocratic rhetoric to justify this inequality, claiming that some people are just better equipped at meeting economic demands due to their intelligence and made the effort to do so. Powerlessness is rebranded as simply unqualified and unintelligent.

### Capitalism and marginalisation

Completing the trifecta of meritocratic oppression is capitalism: a multifactorial, hierarchical, growth-dependent, consumerist-funded, economic system that dominates many, if not all, societies. Capitalism is a free-market economic system driven by profits to increase productivity and economic growth [91, 92]. In a capitalist economy, the economy must grow to raise living standards and well-being [93]. Capitalism thus requires mass consumerism and increased productivity. Increasing productivity is the responsibility of employers and employees to produce more work more efficiently to reduce costs and thereby increase profit margins. The fewer limits to productivity, the more products and services to be provided, the more consumers are satisfied, and the higher profits reaped. Systems *must* be productive. As such, those who have higher productivity, or can increase their productivity, are more valued in capitalist societies. This system marginalises entire groups of people in several ways.

First, capitalism forces individuals to be productive by creating an environment in which they *must* compete with each other for basic goods, like economic stability and independence [94]. This is the manifestation of oppression in the form of marginalisation. Young [62] argues that the productivity requirement of capitalism creates an underclass of people permanently confined to lives the system of labour cannot or will not use: “Whole categories of people are expelled from useful participation in social life”. Young exposes how the elderly through forced retirement or being laid off from employment for being too aged, single mothers from work due to their unfair and highly demanding parental responsibilities, women from traditionally masculine employment, certain racial groups from work traditionally reserved for white people, and those with disabilities from work in general (regardless of whether their condition directly affects the type of work). Capitalism legitimises this exclusion on utilitarian grounds: the only way capitalism can improve standards of living is if productivity increases, so thereby everyone is better off by using productivity measures even if this excludes some.

Second, this marganilsation creates an imbalance of power whereby those who cannot work must rely on social welfare. Young [62] argues that those marginalised from work are forced into dependency on arbitrary systems for welfare. They must give up their rights, freedoms, privacy, and individual choice to access welfare, must lay bare their most intimate details and obey the strict and degrading systems in place simply to survive; they lack any choice or autonomy in the matter. The system is self-reinforcing: capitalism excludes the unproductive and justifies their exclusion on the grounds that it must now increase economic growth to afford the welfare the unproductive require, which ultimately requires the employment of the more productive. Capitalism creates an imbalance of power whereby those who cannot work must rely on social welfare.

Third, Young (1990, 54) argues that the social exclusion marginalised groups suffer is dangerous because they are denied “the opportunity to exercise capacities in socially defined and recognised ways”. Doing something of meaningful value within society allows people to be socially included and *needed.* But in a meritocracy, a lack of need is seen as a lack of merit. The unproductive are thus “victims of material deprivation, social exclusion, isolation, and the affective repercussions of these such as ‘low self-worth” [62]. MacKay [53] argues that because “most of society’s productive and recognized activities take place in contexts of organized social cooperation”, e.g., education and employment, exclusion from these activities “involves unjust deprivation of cultural and practical opportunities for exercising capacities, being recognized as a person, and interacting with others”. Migone [95] recognises that marginalisation increases income inequality and argues that this “amounts to restricting the “capabilities” of all but high-income individuals.” Capabilities are what Sen [96] defines as a person’s “actual ability to achieve various valuable functionings as a part of living”. Excluding the unproductive from the opportunity to exercise their capabilities to achieve the parts of a person that enable them to lead a life at all is an unjust form of disadvantage and oppression [96]. But in a meritocratic society, the unproductive are often blamed for their exclusion, and Migone [95] argues that they are “looked on with suspicion if not contempt” which exacerbates the oppression they suffer. This oppression is pervasive, pernicious, and reinforcing: there is little those who lack privilege can do but conform to its norms.

## Oppression and CE

CE provide an opportunity to escape these forms of oppression. If a person had to choose between economic and social imperialism, marginalisation, or powerlessness or cognitively enhancing themselves (or their offspring), it seems that there is simply no option. If we faced a lifetime of exclusion from society, from meaningful work, from economic independence, autonomy, and financial freedom, then there is almost no choice; we would also be cognitively enhanced to be as productive and intelligent as we could possibly be. We would enhance our intelligence and any other traits that keep us focused and alert. Ray [97, 98], for example, has argued for the use of study drugs for disadvantaged school students to improve their education outcomes and equalise their opportunities. Veit and Browning [99] have similarly argued for enhancements for women to better manage the unequal proportion of domestic work and employment they endure in patriarchal societies. But, as Elliot [11] argues, the need for enhancements becomes objectionable when the problems such enhancements fix have objectionable social roots: “Many people have soul-deadening jobs that require them to spend long periods of time staring at a computer screen performing boring mental tasks. It is not hard to see why they are interested in stimulants. But the larger problem is their boring soul-deadening jobs.” So, what does this mean for the autonomy of those pursuing CE, in any form?

My first claim is that choosing to conform to oppression by using CE to be less powerless, dominated, or marginalised, is not an authentic choice. Forced choice is a form of external manipulation rather than authentic desire [54]. Capitalism has a grip on our bodies. It forces us to modify our bodies to be maximally productive so that we can beat our competition and stay economically afloat. But in doing so, we are not the authentic controllers of the form our bodies take. Foucault [100] discusses the modification of the body to conform to societal control in the context of the concept of the “solider” being formed from military disciplinary regimes:

in every society, the body was in the grip of very strict powers, which imposed on it constraints, prohibitions or obligations… A “political anatomy,” which was also a “mechanics of power,” was being born; it defined how one may have a hold over others’ bodies, not only so that they may do what one wishes, but so that they may operate as one wishes, with the techniques, the speed and the efficiency that one determines.

McKenny [101] argues “the result is that we express how concerned for the buttery vulnerability of the other by seeking to ensure that his or her body measures up to norms designed to render our bodies maximally productive, useful, and efficient.” As such, Winkler [102] argues that the locus of concern in the face of such oppression becomes the body, “the individual is exhorted to turn to bodily control and enhancement as a response to social or political problems.” CE provide us with a means to escape an oppressive economy. This is, as Kraemer defines, “free choice under pressure” [103], or a form of bargaining with one’s oppression [104]. This environmental manipulation undermines our authenticity; in trying to avoid the consequences of oppression, such as powerlessness and marginalisation, we modify our bodies with CE. We use them out of fear, not freedom.

My second claim is that the universal value for CE that increase our abstract rational intelligence is not a cultural norm but cultural imperialism. The default notion of intelligence as the white masculine is an inauthentic preference due to psychological manipulation. Many of us internalise this culturally imperialist notion of intelligence as the measure of human worth. Sandel [63] argues that the years of anxious striving in school to get into prestigious colleges and to get degrees leaves young people vulnerable with fragile self-worth and their self-esteem contingent on achievement. In failing to do well on imperialist standardisation tests, receive credentials, or be economically productive, we become vulnerable to socio-economic rejection, and our self-authorisation as competent, valuable, and meaningful human beings is corroded [44]. The dominant belief that the capitalist and credentialist meritocracy is the measure of verified and objective merit undermines the self-worth and self-trust of people in themselves as valuable persons. Meritocratic norms of intelligence are a universal currency of social value and rank, and so the pursuit of superintelligence through CE that aligns with this universal oppressive norm is not necessarily the intrinsic value for intelligence broadly, but the inauthentic adaptation to preference and value oppressive norms. With the many kinds of intelligence available to value, when someone pursues CE (whether because they value this type of intelligence or are seeking the benefits of such intelligence) is not a coincidence, but the collective result of oppression. That is, people are inauthentically pursuing these goods due to the psychological and environmental effects of oppression, rather than due to the intrinsic worth of this narrow type of intelligence.

A worry might arise here, that I am arguing that those who pursue CE are lacking authenticity and therefore autonomy *altogether*. I would first reply to such a concern that the societal pursuit of a very specific cognitive intelligence that has been privileged by racist and patriarchal systems should make us question the inauthenticity in those pursuing such intelligence. This is not to say that people cannot genuinely and authentically want to improve this type of intelligence, but that such people are far rarer than proponents of CE give them credit for. Many, if not most, of us are not pursuing such goals authentically. Furthermore, even those making an authentic decision must nevertheless grapple with the moral consequences of their decision. Taking advantage of oppressive norms reinforces the oppression. As Grant [59] argues, we have moral duties to resist oppressive norms, because every act of non-cooperation to resist oppressive norms makes it *more* difficult for those suffering from oppressive norms to resist them, and can even encourage or co-op others to similarly exploit them. To benefit from being in the privileged class in an oppressive society is to *depend* on the oppression and subsequently reinforce such norms.

I would also respond to concerns by pointing out that people who have adapted preferences to oppressive contexts do not lack rationality or agency, nor are they cultural dopes or morally weak for failing to resist oppression [105]. Identifying a lack of authenticity or autonomy does not mean a lack of agency altogether. But how do we marry a lack of autonomy with respecting autonomy when an agent cannot escape an oppressive environment? Khader [106] calls this the agency dilemma: what are the norms around identifying someone’s difficult or vulnerable situation in response to their oppression whilst recognising and respecting their agency? Khader [106] does so by distinguishing between appropriately and inappropriately adapted preferences, namely, preferences inconsistent with basic flourishing formed under conditions nonconductive to basic flourishing who “seem to be complicit in perpetuating their own oppression and deprivation”. Khader acknowledges that in oppressive contexts, there are often strong social and economic incentives for people to make self-harming decisions in order to achieve higher order goals, such as safety and security. CE may therefore not be the promised beacon of human superintelligence ready to light the way toward a bright and shiny post-enhanced world, but the result of a motivational structure governed largely by oppressive norms that protect economic growth rather than human flourishing [49].

## Intelligence as intrinsically valuable

A response might be made that intelligence is intrinsically and objectively valuable for human well-being [36, 107]. Being intelligent is a prerequisite for obtaining knowledge, which is often a central aspect of objective theories of well-being [108–111], and autonomy, which is the central aspect of subjective theories of well-being happiness [112]. For example, Schaefer et al. have argued that enhancements can improve human capacities such as deductive/logical competence, pattern recognition, linguistic ability, memory, comprehension, and critical analysis [8]. They argue that these traits improve a person’s capability for autonomy, and autonomy is not instrumentally, but intrinsically, valuable. As such, we have objective-given reason to value CE, regardless of the ways in which intelligence is also positionally valuable or oppressively measured. Some might even argue that the value of productivity is simply a defensible pluralist cultural value of improvement and efficiency. Indeed, we would be mistaken and likely unsuccessful in an attempt to disprove the objective value of the capacity for rationality, and the essential role it plays in making and achieving life plans and exercising our autonomy [6–8]. With the claim that intelligence is essential for autonomy and well-being, I have no criticism. What I criticise is that subsequent claim that *more* intelligence is intrinsically valuable.

More is not always better. Superintelligence, or enhanced intelligence, as a means to improve human well-being as both an empirical and philosophical claim, is deeply controversial. As an empirical claim, that “smarter” people are “happier” is widely accepted as a pervasive myth [113]. Hauskeller argues: “If someone’s life does not go well it is not usually because they are not (instrumentally) clever enough”. In philosophical literature, Hauskeller [114] points out that possessing intelligence is not itself a mark of whether one has well-being: “if your goals are misguided, being a good rational decision-maker will not get you very far”. Having capacity to think of more options or life plans doesn’t mean those life plans are objectively worthwhile [58], or that we will achieve them easier or act in accordance with reason [115]. Indeed, if we consider intelligence attributes more specifically, such as enhancing memory, we quickly realising that whilst CE might prepare us for a once-off exam or with court preparation, the usefulness of memory is optimally limited: we are better off for forgetting irrelevant details, embarrassing moments, and importantly, traumatic experiences. Enhancing our abstract reasoning might also compromise many other capacities. For example, maintaining healthy relationships requires us to forgive and *forget* some grievances and mistakes. Furthermore, being able to listen to music is, I think, an important part of being a human – this does not mean that we should try to listen to more music at once, or music all day every day. Being able to do math is an important skill for budgeting and managing our home affairs, but this doesn’t mean we need to be able to do complex calculus or quantum mechanics.

The relationship between intelligence, autonomy, and well-being might therefore be best conceived as a goalpost. Intelligence forms the goalposts of well-being and every person, based on who they are and their beliefs, values, and experiences can kick a goal to achieve their well-being as long as they have goalposts. More intelligence does not make those goalposts further apart; it simply shifts the goalposts across the axis. Intelligence is a threshold by which human beings understand the concept of *a* good life, but it is not a measure by which we rank and compare different standards of *the* good life: we cannot compare the well-being brought about by a musician who delights in their music with a professional organiser who delights in their cleaning [116]. A moderately intelligent person kicking a goal through their goalposts can be equally satisfied as someone with high intelligence kicking a goal through theirs.

The focus on CE as a way to be, do, produce, remember, think, and create more, shifts the focus from the human conducting the act, to the outcomes they can possibly achieve, in doing so, we shift the purpose of human capabilities off from the way exercising them produces meaning back to the human, to the way exercising them produces goods. Rick Rubin [117] writes “art is the by-product of creativity”. Things, achievements, art, music, and knowledge are all the by-products of human life, not the goals. People want to *make* music, they want to *create* art, they want to *learn* knowledge, and *play* sports. If the experiment machine taught us anything [118], it’s that humans want to do. They want to do again and again: we reread books and revisit artworks, not to memorise their features so that we never have to experience them again, but to feel and experience creativity. We listen to the same songs, undertake the same hikes, eat the same foods. We do not need enhanced intelligence to achieve any further goodness beyond that which we already experience from participating in cognitive and creative activities as inherently rational creatures. The only enhancement that can actually allow us to *do* more are ones that give us more *time.*

Whilst intelligence can be defined in many non-problematic ways and is intrinsically valuable regardless of how it can also be weaponised by oppressive systems, the nature of genetic enhancements is not to appreciate this trait but to *increase* how much of it a person has. And when we ask why we need more of it, all we can do it point to our environment: I believe most of us need CE to avoid oppression rather than to improve our own authentic desires. That we feel, therefore, that genetic enhancements are valuable because productivity is where humans derive value reveals a cultural bias in which we collectively *measure* our lives according to how *good* we are at producing things. Indeed, when we ask who is benefited by CE, as defined by very narrow concept of intelligence as abstract rational thinking, in a capitalist economy governed by credentials, testing, and merit, we realise the benefits flow toward increased profits and productivity, neither of which are benefits to people [62]. Furthermore, humans do not need to *improve* their output or increase their *efficiency* for their own well-being, they need to do so to improve the economy. Any appeal to CE as a means to increase well-being reinforces the very oppression that defines the unproductive as unremarkable and marginalises them.

## CE already exist, so what’s special about genetic enhancements?

I have, for the most part, considered the impact of oppressive norms systemic to the capitalist meritocracy on the authenticity of those pursuing CE.^12^ What I wish to further consider, is the difference between existing CE and CGEs. I believe there are at least three significant distinctions that render the proposed research and use of genetic technologies more morally concerning than existing methods.

First, CGEs are conceptually distinct to other forms of CE. Genetic modification in accordance with oppressive norms means that a person can physically *embody* their oppressor: their bodies become technoplastic, mouldable to the unjust and discriminatory norms of racist, patriarchal economies. This is substantially, conceptually, and morally distinct, from the temporary and generally non-invasive modifications brought about by sipping coffee, taking a drug, or even using brain-computer interfaces whilst a person studies or works. We need not accept genetic determinism and reject the role of society, relationships, and childhood in developing our cognitive functioning, to accept that *building* or *shaping *a person who better conforms with oppressive norms is morally problematic. We would become the GMO corn of the capitalist meritocracy: we would serve economy well, but at what cost to human well-being, diversity, and meaning in life.

Second, genetic technologies do not yet exist. Unlike the coffee industry, the consumer market for BCI’s, and even the off-market use of pharmaceuticals like Ritalin, the regulation and response to genetic technologies is fundamentally different. We do not have to pull back existing technologies or deprive people of their existing practices. Indeed, genetic modification requires a team effort with expensive equipment and scientific facilities: regulating its implementation is vastly different to controlling illegal market drug use or the approval of consumer BCI’s. This is not to say that we ought to *ban* all genetic modifications, but that insofar as it can only be provided in the assisted reproductive technology industry may make it far more feasible, and therefore reasonable, to proactively regulate. Furthermore, that such means of improving one’s cognitive functioning exist, does not infer that any and all methods of achieving the same outcome are similarly justified. The ends do not always justify the means, nor in this case in which the means are conforming to oppressive norms, are the means always justified.

Third, given that CGEs are more likely to be successful when performed on embryos, such technology will be implemented at the request and consent of parents for their offspring. Whilst we acknowledge that parents generally have autonomy to act in the child’s best interests, we might respond that the beneficiary of the CE is not necessarily the child, but the economy. Even acknowledging that children might benefit from the increased social and economic benefits of being rationally superior to their peers, this would require permitting parents to modify their child in accordance with very specific, racist, and gendered ideas of what kind of intelligence is best. As well as to reinforce oppressive norms that other children, who are not enhanced, will suffer. After all, genetic enhancement is the improvement of intelligence on an already cognitively healthy child. As such, we should be concerned that there is universal, and nonautonomous desire for CE that so deeply pervades social consciousness that we would sooner genetically modify our children in accordance with racism, sexism, and capitalism, than address the systems themselves.

## Conclusion

CGEs are a worry: our desire for them is likely only the product of our societal struggle to survive in an oppressive system built on racist and patriarchal norms of intelligence. After all, so much of our lives depends on simply how smart we are according to a very narrow set of tests. Many of us have also internalised these meritocratic norms; judging ourselves, and others, as praiseworthy or blameworthy for our respective incomes, positions, jobs, and lives. To put it simply, we lack autonomy to determine what types of intelligence really do matter the most: such decisions are made for us. As such, the moral permissibility of researching, developing, and implementing such technology lacks grounding on the basis that people autonomously value, and use, such enhancements. Indeed, we have also indirectly argued that such enhancements are not necessarily intrinsically good for us, only insofar as they allow us to survive in an oppressive environment.

So, what do we do about it? Scholars, in particular bioethicists, need to recalibrate debates around the ethics of CE to consider the ways in which we are oppressed into *needing* such technologies, rather than autonomously valuing them [122]. In normative research, scholars analysing the overall moral permissibility of these enhancement technologies must weigh the negative impact of reinforcing an oppressive norm against the proposed benefits and acknowledge that many of these benefits are positionally important in an oppressive society, rather than intrinsically beneficial to human wellbeing. The moral weight of the benefits of genetic enhancements may be less significant in light of the view that we do not autonomously pursue them: what good are they for us, if after all, we do not pursue them for own good? In empirical research, scholars must warn participants of the effects of oppressive norms in undermining their autonomous values, as well as study public perceptions of the value of CE as a product of an oppressive system rooted in patriarchal and racist norms of intelligence [119, 120].

The research produced from these endeavours is important for developing morally robust regulatory approaches to both existing forms of CE, as well as future technologies such as CGEs. Whilst I will not undertake an analysis of the most optimal way to regulate CE – the use of CE is simply too widespread despite generally prohibitive or restrictive regulatory approaches – I will briefly suggest that any attempt to regulate the current uses of CE ought to be sensitive to the oppressive norms that perniciously undermine the autonomy of those who seek to use them, particularly those desperate to use them illegally. More specifically, in light of my claims about the embodied nature of CGEs and the way such technologies will be sought by parents for future children through assisted reproductive technology processes, I endorse scholars to research how guidance and restrictions ought to be employed to regulate and prevent persons inauthentically pursuing oppressive norms through genetic enhancement. I believe that insofar as this is an issue with authentic preference and autonomy, a relational, rather than punitive or restrictive, approach would best guide us toward a less oppressive system.

For those of us who, whilst not yet faced with CGE, are faced with the pressures that judge and undermine the self-respect and self-trust in our own intelligence, I endorse Clare Chamber’s [121] reflective questions:

…to what extent are your actions shaped by or reacting to dominant social norms? How are you acting autonomously in how you push towards this modification? Is this modification necessary to gain social status or acceptance? Are you pursuing this modification as the result of socially induced shame? Do the social pressures that you’re reacting to fit within structure of norms and institutions that undermine your equality? Does the modification that feels necessary to harm or enhance your health? Will being modified make you feel better? What is the evidence for that?

## Data Availability

Data sharing is not applicable to this article as no datasets were generated or analysed during the current study.

## Footnotes

1 DeGrazia presents the traditional views of authenticity as rooted in theories of identity in analytic philosophy, namely, narrative identity or numerical identity. Numerical identity is generally conceived as a form of *psychological* continuity of memory or consciousness, as endorsed by Parfit [2] and Locke [3]. Although some less stringent theories of psychological continuity only require continuity of basic psychological capacities i.e., McMahan [4]. Numerical identity can also be conceived as *biological* continuity, which allows us to keep separate the metaphysical nature of identity from the philosophical task of identifying what is valuable about our existence [1]. Numerical identity is helpful in answering questions such as whether one being at one time is the same as the being at another time. *Narrative* identity is a subjectively relative concept of identity; it asks whether the person identifies with themselves and their conception of themselves. This is a value-laden question and as DeGrazia argues, “inherently psychological”. DeGrazia applies the enhancement question through a narrative lens, to argue that people can legitimately and authentically change their self-conception through enhancements.

2 A meritocracy is not inherently oppressive, however, many of our meritocratic systems *are* because, and I argue that, the norms and values by which we measure merit are those from oppressive systems, such as racism and patriarchy.

3 Whilst the scope of this paper is limited to cognitive-gene enhancements, some scholars have considered the relationship between authenticity and mechanical enhancements, such as deep brain stimulation. See, for example, Pugh, Maslen, and Savulescu [19].

4 See, for example, Bognar [30], Barclay [31], Carapeto Raposo [32], and Prince [10].

5 See, for example, Sandel [33], Singer [34, 35], and Savulescu [36].

6 This is because I am not concerned with the decisions of those who lack capacity to consent to using cognitive-gene enhancements. In such cases, someone else with legal guardianship who has capacity will make such decisions. I am also not concerned with cases in which a person lacks sufficient information about the benefits and risks of using CE. For the sake of this paper, I will presume individuals are receiving sufficient information prior to purchasing or using such devices. Although, analysing the informational understanding of those using cognitive enhancers would be rich grounds for research.

7 The relationship between autonomy and authenticity depends on which definition of each concept we use. But for the purposes of this paper, I endorse a relational view of autonomy which *includes* authenticity requirements. Authenticity is necessary, although not sufficient for autonomy. As such, if a person is not making an authentic decision, I argue they are also not making an autonomous decision. This is separate from whether they are exercising their agency, which I will address later in this paper.

8 Relational theories of autonomy are often pitted against liberal notions of autonomy. But for the purposes of this paper, I would like to highlight how relational theories can richly illuminate what it means to be authentic and make a realistically voluntary decision.

9 There are different names for the act of affirming one’s decision-making process in the literature. For example, Brucker [45] defines this as asking whether a person retains their adapted preference after critical reflection. McLeod [46] defines this as ‘reflective endorsement’: when a person endorses the ideal and the value-forming process. DeGrazia [47] states that influences are illegitimate when the person ‘would, on careful reflection, consider alienating’, see also, MacKay [48], Mackenzie [49], and Christman [50], for the concept of alienation in determining authenticity. Westlund [51] considers this ‘dialogical answerability’ in which we become responsible for our values through a ‘rigorous process of critical answerability’. Feinberg [52] calls this the ongoing process a ‘habit of critical self-revision’.

13 I have explored the relationship between oppression, autonomy, and genetic cosmetic enhancements elsewhere [122], where I have argued that patriarchal beauty norms undermine women's self-respect and self-trust, as well as the meaningful options available to them, thus undermining the autonomy of women who pursue cosmetic enhancements. 

10 When I use the terms ‘masculine’ and ‘feminine’, I am referring to the social constructs that define gender, and the norms and characteristics that have been historically and culturally associated with such constructs.

11 This critique addresses societies that endorse standardization testing rooted in predominantly white and patriarchal norms of knowledge despite having diversity across its population in both race and gender. A response might be considered here that some societies are more homogenous than others, for example, predominantly white countries may be justified in standardizing their tests in accordance with their homogenous populations. However, predominant doesn’t mean exclusive and it is outside the scope of this paper to consider how education ought to be restructured to avoid cultural imperialism. Nevertheless, to such countries, my feminist critique of the patriarchal standardization of knowledge will also generally apply. Furthermore, for want of space and scope, I have not consulted the rich perspectives within disability scholarship that would be applicable here, and I endorse future research applying my critique in other ways. For example, I think this analysis would also benefit from those with expertise in neurodivergence in the context of how children and students learn and the ways in which enforcing standardised teaching methods on a diverse group of students with diverse learning needs can discriminate against those outside the norm.

12 I wish to briefly acknowledge here that if society was just and did not oppressively discriminate against other types of intelligence or work, then a person who uses genetic enhancements would likely be authentically doing so. My thesis is practically contingent on existing oppressive norms and structure, rather than inherently critical of this type of intelligence. Nevertheless, persons pursuing CE in a just society would still be subject to other moral arguments. For example, are those who are using cognitive enhancement, even if society was just, justified in pursuing any positional advantage at the cost of others?
